# Infants’ advances in speech perception shape their earliest links between language and cognition

**DOI:** 10.1038/s41598-019-39511-9

**Published:** 2019-03-01

**Authors:** Danielle R. Perszyk, Sandra R. Waxman

**Affiliations:** Northwestern University, Evanston, USA

## Abstract

The power of human language derives not only from the precision of its signal or the complexity of its grammar, but also from its links to cognition. Infants as young as 3 months have begun to link language and core cognitive capacities. At 3 and 4 months, this link is not exclusive to human language: listening to vocalizations of nonhuman primates also supports infant cognition. By 6 months, infants have tuned this link to human speech alone. Here we provide evidence that infants’ increasing precision in speech perception shapes which signals they will link to cognition. Infants listening to German, a nonnative language that shares key rhythmic and prosodic properties with their own native language (English), successfully formed object categories. In contrast, those listening to Cantonese, a language that differs considerably in these suprasegmental properties, failed. This provides the first evidence that infants’ increasingly precise perceptual tuning to the sounds of their native language sets constraints on the range of human languages they will link to cognition: infants begin to specify *which* human languages they will link to core cognitive capacities even *before* they sever the link between nonhuman primate vocalizations and cognition.

## Introduction

Language is a signature of our species. It enables us to express the contents of our own minds and to influence the minds of others. Human language is unparalleled in the precision of its signals and the complexity of its grammar, but the enduring power of language also derives from its inextricable links to cognition^1^. Incredibly, infants as young as 3 months of age are well on their way to establishing such links^2–4^. Most of the evidence for this precocious link comes from a simple yet powerful task designed to measure preverbal infants’ success in forming object categories, a core conceptual process that provides a groundwork for establishing meaning^2,3,5–7^. In the classic categorization task, infants first view a series of “familiarization” images from one object category (e.g., dinosaurs). Then they simultaneously view two new “test” images: a new exemplar from the familiarized category (e.g., another dinosaur; “familiar object”) and a new exemplar from a novel category (e.g., a fish; “novel object”). Infants’ ability to distinguish between the familiar and novel test images, measured by looking times, indicates whether they have formed the object category.

Crucially, infants’ success in this cognitive task depends upon the auditory information that accompanies the visual images presented during familiarization. When each image is paired with a segment of human language, infants as young as 3 months successfully form object categories; when the very same images are paired with tone sequences or a segment of backward speech, infants fail to do so^2,8^. But language is not the only signal that boosts infant cognition at 3–4 months: listening to the vocalizations of nonhuman primates (*blue-eyed Madagascar lemur; Eulemur macaco flavifron*) also supports object categorization^8^. This suggests that at 3–4 months, infants have a broad initial template that permits them to link the vocalizations of both humans and nonhuman primates to core conceptual capacities. However, the cognitive advantage conferred by listening to nonhuman primate vocalizations is short-lived: by 6 months, nonhuman primate vocalizations no longer support object categorization^8^.

This evidence offers new insights into the developmental mechanisms by which infants begin to establish an abstract link between language and cognition. After all, infants as young as 3–4 months of age do not yet parse individual words from the speech stream and assign them to visual referents (e.g., the visual images presented in the categorization task). Instead, very young infants must rely upon another, perhaps simpler mechanism to initiate the link to cognition. For example, simply listening to language may enhance 3- and 4-month-olds infants’ attention or arousal, and in this way boost core cognition^9^.

In addition to providing insights into the ontogenetic foundations for human language and its links to cognition, these findings raise a vital new question: Does infants’ precocious language-cognition link interface with their rapidly increasing precision in processing the human speech signal in the first months of life? Decades of research document infants’ increasing specialization for processing their native language in the first months of life. At birth, newborns distinguish broadly among human languages on the basis of rhythmic class (cf. stress-timed, syllable-timed, mora-timed)^10^. Moreover, the cries that newborns produce mirror the broad prosodic contours of their native language^11^. By 2–3 months, infants tune increasingly to the sounds of their language and eventually discriminate their own native language (cf. English) from other languages of the same rhythmic class (cf. Dutch or German)^12^. Considerable neural and behavioral evidence reveals that rhythm and prosody are instrumental as infants tune increasingly to the sounds of their own native language^10,13–16^. Moreover, evidence suggests that infants’ native language comes to serve as a “perceptual magnet” (cf.^17^), shaping infants’ perception of the sounds of native and nonnative languages.

But what remains unknown is whether and when infants’ exquisite perceptual tuning to their own native language interfaces with the more abstract link between language and core cognitive capacities, like object categorization. Here, we test the hypothesis that infants’ perceptual tuning to their native language in the first months of life^10–16,18,19^ has consequences beyond speech perception alone, setting constraints upon *which* language(s) they will link to core conceptual capacities. Does infants’ increasing sensitivity to various acoustic features of language—namely, those that demark their native language—guide them to link some languages, but not others, to core conceptual processes?

To address this question, we considered English-acquiring 3- to-4-month-old infants’ responses to one of two nonnative languages (German or Cantonese) in the context of the classic object categorization task. We selected German (Experiment 1) because it is a language that is phonologically ‘near’ to English; these two languages share key rhythmic (e.g., both have been classified as stress-timed) and suprasegmental, prosodic features^20^. We selected Cantonese (Experiment 2) because it is a language that is phonologically more ‘distant’ from English; Cantonese differs from English in rhythm (e.g., it has been classified as syllable-timed) and several other suprasegmental, prosodic features^21^. We reason as follows. If infants’ advances in speech perception do not yet interface with the still-emerging link language-cognition link, then listening to any human language should boost infant cognition. Alternatively, if infants’ rapid tuning to their native speech does interface with their emerging link to core cognition, then a more nuanced pattern should emerge: listening to German should boost infant cognition, yet listening to Cantonese should have a weaker effect, if any. Notice that this would constitute evidence that the developmental trajectory underlying infants’ precocious link between language and cognition unfolds along a different path than that underlying the link between nonhuman primate vocalizations and cognition.

## Results

### Does listening to German boost infant categorization?

**Figure 1:**
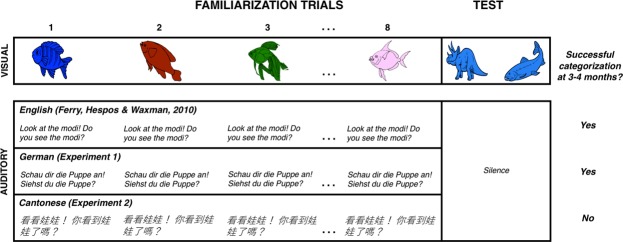
Experimental design and results. During Familiarization, each infant viewed 8 distinct visual images (20 s each), presented sequentially. In a previous study, each image was accompanied by a segment of infant-directed English^2^. In the current studies, each image was accompanied by a segment of a nonnative language produced in an infant-directed speech register (German, Experiment 1; Cantonese, Experiment 2). For each familiarization image, this segment was presented twice. At test (20 s), each infant viewed 2 images, a new member of the now-familiar category and a member of a novel category. These were presented simultaneously in silence.

In Experiment 1, infants participated in the object categorization task; each familiarization image was presented in conjunction with a segment of infant-directed German. Infants’ performance at test revealed that they successfully formed the object categories, favoring the novel over the familiar object at test (Fig. 1). This novelty preference was robust at both the group (*M*_*novelty preference*_ = 0.59, *SD*_*novelty preference*_ = 0.13; *t*(30) = 3.7, *p* < 0.001, Cohen’s *d* = 0.69) and individual level (*Z* = 1.98, *p* = 0.048, sign test). There was no correlation between infants’ age and their test performance (*r* = 0.174, *p* = 0.324).

### Does listening to Cantonese boost infant categorization?

In Experiment 2, infants participated in the same object categorization task as in Experiment 1, but this time each familiarization image was presented in conjunction with a segment of infant-directed Cantonese. Although these infants devoted the same amount of attention to the familiarization objects as their counterparts listening to German (*p* > 0.05), they failed to form object categories, showing no preference for either the novel or familiar object at test (*M*_*novelty preference*_ = 0.51, *SD*_*novelty preference*_ = 0.15; *t*(37) = 0.38, *p* = 0.71, Cohen’s *d* = 0.067) (Fig. 1). Their chance-level test performance stands in sharp contrast to the success of their age-mates listening to either German, English^2^, or nonhuman primate vocalizations^8^ at 3–4 months. Infants’ performance at test did not correlate with their age (*r* = 0.085, *p* = 0.608). In addition, the test preferences of infants listening to Cantonese were significantly lower than those listening to German, *t*(66.6) = −2.3, *p* = 0.025, Cohen’s *d* = 0.57.

## Discussion

These results advance our understanding of the origins of a uniquely human link between language and cognition. First, they reveal that infants’ rapid tuning to the sounds of their native language has an impact beyond speech perception alone: it serves as an anchor as infants forge their earliest links between human language and cognition. At 3–4 months, English-acquiring infants listening to German, a language that shares rhythmic and other suprasegmental features with English, successfully form object categories. In contrast, listening to Cantonese, a language from a different rhythmic class and exhibiting different suprasegmental features, confers no such cognitive advantage. This reveals that as early as 3–4 months, infants’ advances in speech processing play a central role in their establishment of a language-cognition link. In addition, this evidence is consistent with claims that rhythm may have likewise played a central role in the evolution of human speech^22,23^.

Second, infants’ responses to the two nonnative languages tested here, coupled with their previously reported responses to vocalizations of nonhuman primates^8^, show that infants begin to specify *which* languages they will link to core cognitive capacities even *before* they sever the link between nonhuman primate vocalizations and cognition. Although this pattern may appear paradoxical, it converges with neural and behavioral evidence indicating that infants begin tuning precisely among human vocalizations before they tune out nonhuman vocalizations^18,24^.

This developmental pattern also converges well with developmental evidence concerning the dedicated face processing system in humans. Newborn infants successfully distinguish among individual faces of both humans and nonhuman primates^25^. By 3 months, they exhibit perceptual tuning in their responses to human faces: their perception of faces of individuals from their own race (like their perception of speech sounds from their native language) is enhanced even as their perception of faces of individuals from other racial groups is diminished (a phenomenon often described as the “other-race effect”)^26,27^. However, during this same developmental window, they maintain their precision in distinguishing among faces of individual nonhuman primates^28^. Certainly, this parallel between the development of dedicated systems for face and for speech processing is intriguing. Notice, though, that the evidence we report here goes beyond perceptual tuning (to faces and voices) alone. To the best of our knowledge, this is the first evidence that infants’ perceptual tuning to their native speech signals has consequences for which signals—here, which languages—they will link to cognition.

Third, infants’ responses to a nonnative language (German) that is phonologically close to their own native language (English), coupled with their responses to their own native language (English)^2^, reveal that listening to both languages supports infant cognition, There is, however, a subtle difference in infants’ responses to these two languages at 3 months. Prior work established that infants listening to their native language exhibit a systematic shift in their expression of object categorization, preferring the familiar test image at 3 months, but the novel test image at 4 months and beyond^2,5,6^. Shifts like this, from familiarity to novelty preferences, are common in infant research^2,8,29–39^ and are thought to reflect developmental advances in infants’ efficiency in processing the stimulus materials^2,8,40–43^. Importantly, since both familiarity and novelty preferences reflect an ability to distinguish between the familiar and novel test images, both are taken as evidence of successful categorization.

But if infants listening to their native language shift from familiarity to novelty preferences between 3 and 4 months, why do those listening to German reveal novelty preferences at both 3 and 4 months? We suspect that this may be a consequence of the processing resources that very young infants devote when listening to their own native language: these infants, so actively engaged in tuning to the sounds of their own native language, may devote greater attentional resources to processing their own native language(s) than others. If this is the case, it would reduce the remaining cognitive resources available for processing the visual images in our categorization task. In future work, it will be possible to test this hypothesis by examining neural indices of attention and arousal in infants as they listen to their native and nonnative languages.

This work also opens several new avenues for research. For example, it will be important to extend the range of human languages and the range of language-learners examined. Another open question concerns the role of exposure. We know that infants adapt flexibly to the sounds of new languages (for review, see^44^). What remains unknown is whether exposing infants to naturalistic speech of nonnative, phonologically ‘distant’ languages (e.g., exposing English-acquiring infants to Cantonese and Cantonese-acquiring infants to English) is sufficient to enable these infants to link them to core cognitive capacities^45^.

Finally, this work offers new insights into how the uniquely human link between language and cognition may have emerged in evolution. Infants’ responses to the two nonnative languages tested here, considered in conjunction with their responses to English^2^ and to the vocalizations of nonhuman primates^8^, are consistent with the possibility that infants’ earliest links to cognition stem from two distinct systems—only one of which is dedicated specifically to human speech^18,46–49^. Perhaps listening to human languages, both native and nonnative, engages infants’ dedicated speech processing system, and infants’ exquisite tuning to their native language sets constraints upon which among these languages they will link to cognition. During this same developmental window, vocalizations of nonhuman primates may boost infant cognition by engaging another system, one not specialized for speech.

This possibility aligns with recent neuroanatomical evidence^46,47^ demonstrating that in humans, a cortical system for processing and producing articulate speech evolved atop a subcortical system that is shared among primates for processing and producing affective vocalizations. The neural reorganization that undergirds human infants’ rapid tuning to native speech may incidentally connect brain regions involved in speech processing with those involved in core conceptual processes. That is, human infants’ exquisite tuning to the speech signal and to other social communicative cues^50,51^ may recruit neural systems implicated in other cognitive processes, serving as a gateway for establishing increasingly precise and abstract links to meaning. (Interestingly, there is no evidence that nonhuman primates undergo such perceptual tuning)^17,52^. Indeed, infants that show greater neural commitment to the sounds of their native language experience more rapid language learning^53^. By extension, this interpretation would suggest that over evolutionary time, the neural reorganization underlying our ancestors’ increasing perceptual-motor precision with human speech likewise served as a gateway for establishing increasingly precise and abstract links to meaning.

## Methods

### Experiment 1

#### Participants

Thirty-one infants recruited from the Greater Chicago Area were included in the final analyses (19 females, *M*_*age*_ = 4.03 months, *SD*_*age*_ = 0.609 months). Experiments were performed with approval and under the accordance of the relevant guidelines established by the Institutional Review Board at Northwestern University. We obtained informed consent from infants’ caregivers at the beginning of each lab visit, and all participants received a book and a toy as compensation for participation. Another 19 participated but were excluded from analyses due to fussiness (7), insufficient attention during familiarization (4), looking exclusively to one test image (2), or a test score more than 2 mean absolute deviations from the mean (6).

#### Materials

Visual stimuli: Line-drawn images of dinosaurs and fish formed two 8-item familiarization sets and two test pairs. Within each familiarization set, images varied in color; within each test pair, images were matched in color. Images were projected onto a white screen.

Auditory stimuli: A segment of infant-directed German speech spoken by a female adult (~5 s, *M*_*pitch*_ = *274 Hz, Range*_*pitch*_ = *130–500 Hz*) played from a hidden speaker below the screen.

#### Procedure

Infants were seated on a caregiver’s lap facing a screen. The visual images were projected onto the screen. Caregivers, who wore opaque glasses, were instructed not to talk to their infants or influence their attention in any way. Infants’ responses were recorded by a video camera hidden below the screen.

Familiarization phase: Visual stimuli (either 8 distinct dinosaurs or fish) were presented on alternating sides of the screen (20 s each). The left/right position of the first familiarization image was counterbalanced across infants. Acoustic stimuli were presented as each image appeared and were repeated 8 s later.

Test phase: Two images appeared side-by-side, in silence, and remained visible for 20 s. The left/right position of the test images was counterbalanced across infants.

#### Coding

Infants’ looking time at test served as our dependent measure. Infants’ left-right eye gaze directions were coded frame-by-frame by trained coders blind to the hypotheses. A second independent observer re-coded 25% of infants. Reliability between observers was high (*r* = 0.93).

### Experiment 2

#### Participants

Thirty-eight infants recruited from the Greater Chicago Area were included in the final analyses (18 females, *M*_*age*_ = 4.07 months, *SD*_*age*_ = 0.60 months). Experiments were performed with approval and under the accordance of the relevant guidelines established by the Institutional Review Board at Northwestern University. We obtained informed consent from infants’ caregivers at the beginning of each lab visit, and all participants received a book and a toy as compensation for participation. Another 15 participated but were excluded from analyses due to fussiness (2), insufficient attention during familiarization (9), looking exclusively to one test image (3), or a test score more than 2 mean absolute deviations from the mean (1).

#### Materials

Identical to Experiment 1, except the auditory stimuli was a segment of infant-directed Cantonese spoken by a female adult (~5 s, *M*_*pitch*_ = *308 Hz, Range*_*pitch*_ = *229–473 Hz*).

#### Procedure

Identical to Experiment 1.

#### Coding

Identical to Experiment 1. A second independent observer re-coded 25% of infants. Reliability between observers was high (*r* = 0.98).
